# Changes in soluble LDL receptor and lipoprotein fractions in response to diet in the DIETFITS weight loss study

**DOI:** 10.1016/j.jlr.2024.100503

**Published:** 2024-01-19

**Authors:** Ronald M. Krauss, Lois M. Fisher, Sarah M. King, Christopher D. Gardner

**Affiliations:** 1 Departments of Pediatrics and Medicine, University of California, San Francisco, San Francisco, CA, USA; 2 Department of Medicine, University of California, San Francisco, San Francisco, CA, USA; 3 Department of Pediatrics, University of California, San Francisco, San Francisco, CA, USA; 4 Stanford Prevention Research Center, Department of Medicine, Stanford University Medical School, Stanford, CA, USA

**Keywords:** triglyceride, VLDL, LDL, lipoproteins/metabolism, cholesterol, nutrition

## Abstract

Circulating levels of the soluble ligand-binding ectodomain of the LDL receptor (sLDLR) that is proteolytically cleaved from the cell surface have been shown to correlate with plasma triglycerides, but the lipid and lipoprotein effects of longitudinal changes in sLDLR have not been examined. We sought to assess associations between changes in sLDLR and detailed lipoprotein measurements between baseline and 6 months in participants in the DIETFITS (Diet Intervention Examining The Factors Interacting with Treatment Success) weight loss trial who were randomly assigned to the low-fat (*n* = 225) or low-carbohydrate (*n* = 236) diet arms. sLDLR was assayed using a proteomic procedure, lipids and apoprotein (apo) B and apoAI were measured by standard assays, and lipoprotein particle subfractions were quantified by ion mobility methodology. Changes in sLDLR were significantly positively associated with changes in plasma cholesterol, triglycerides, apoB, large-sized and medium-sized VLDL, and small and very small LDL, and inversely with changes in large LDL and HDL. The lipoprotein subfraction associations with sLDLR were independent of age, sex, diet, and BMI, but all except for large LDL were reduced to insignificance when adjusted for triglyceride change. Principal component analysis identified three independent clusters of changes in lipoprotein subfractions that accounted for 78% of their total variance. Change in sLDLR was most strongly correlated with change in the principal component that was loaded positively with large VLDL and small and very small LDL and negatively with large LDL and HDL. In conclusion, sLDLR is a component of a cluster of lipids and lipoproteins that are characteristic of atherogenic dyslipidemia.

## Graphical Abstract

**Figure:**
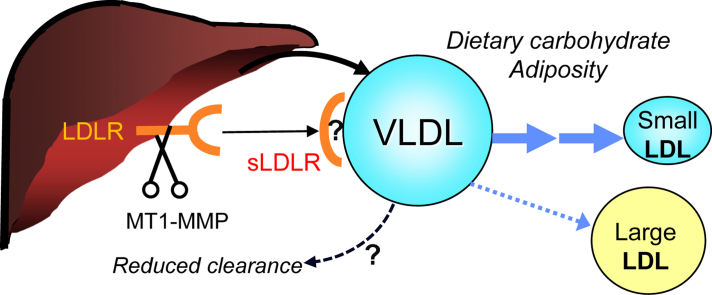

The LDL receptor (LDLR) mediates cellular internalization of lipoproteins containing apoB-100 and apoE by binding these proteins in its ectodomain (1). Hepatic LDLRs are primarily responsible for uptake and subsequent degradation of plasma low LDLs and also contribute to plasma clearance of VLDL and IDL lipoproteins (1). Upregulation of hepatic LDLR expression achieved by increasing activity of the transcription factor SREBP2, for example by treatment with statin drugs, is a major mechanism responsible for lowering levels of these atherogenic lipoproteins and reducing risk of cardiovascular disease (1).

While a high proportion of LDLRs are internalized and recycled to the cell surface following release of their lipoprotein cargoes (1), they can be subject to proteolytic cleavage with release of the soluble LDLR (sLDLR) ectodomain into the circulation (2, 3). A study using cellular and mouse models has shown that this proteolytic step is mediated by membrane type 1-matrix metalloproteinase (MT1-MMP), and that plasma sLDLR and cholesterol levels are reduced, whereas hepatic LDLR is increased in mice lacking hepatic MT1-MMP, with opposite effects of MT1-MMP overexpression (3). Thus, it has been suggested that the pool of cellular LDLR available for lipoprotein uptake is reduced by ectodomain cleavage (3).

Plasma sLDLR levels in humans have been shown to be strongly positively correlated with concentrations of triglyceride, and to a variable extent with LDL-C (4, 5, 6), associations that have been attributed at least in part to physical complexing of sLDLR with apoB- and apoE-containing lipoprotein particles. In a study of children with familial hypercholesterolemia (6), sLDLR levels were not significantly different from those in unaffected controls, but in the combined groups, sLDLR was significantly positively associated with plasma triglyceride as well as large VLDL and small LDL particles, and inversely correlated with large HDL, suggesting a preferential association of sLDLR with the atherogenic dyslipidemia of metabolic syndrome (7). To date, there have been no studies of the effects of dietary interventions on sLDLR or the relation of changes in sLDLR with changes in levels of plasma lipids and lipoproteins.

In the present report, plasma samples from the DIETFITS (Diet Intervention Examining The Factors Interacting with Treatment Success) trial (8) afforded the opportunity to test whether low-carbohydrate (LC) versus low-fat (LF) weight loss diets differentially affect plasma sLDLR levels and to determine the relationships of changes in concentrations of sLDLR with changes in body weight and plasma lipids and lipoprotein particle subfractions.

## Materials and Methods

### Study design

The DIETFITS trial randomized 609 adults aged 18–50 years with a BMI between 28 and 40 and without diabetes to either a healthy LC or healthy LF diet (8, 9). The Stanford University Human Subjects Committee approved the study, which abides by the Declaration of Helsinki principles, and all study participants provided written informed consent. The dietary interventions and their effects on dietary macronutrient composition have been described previously (9). Briefly, the diet protocol included a 1 month run-in period and 22 instructional sessions over 12 months conducted by registered dietitian health educators who were blinded to all laboratory measures. Health educators recommended 60–90 min per day of physical activity and emphasized emotional awareness and behavior modification to support the diet and weight loss program. Weight was assessed at each scheduled visit at the Clinical and Translational Research Unit of the Stanford University. Fasting plasma samples were obtained at baseline and 3, 6, and 12 months. However, plasma sLDLR measurements were obtained only at baseline and 6 months, and thus, all analyses for the present study are based on these two time points. The 6 month interval is sufficient for dietary effects on metabolic measurements to have stabilized and provides greater likelihood of compliance as well as fewer dropouts than after 12 months. Full data were available for 464 participants (supplemental Fig. S1). Two were excluded because of baseline triglyceride levels >400 mg/dl (628 and 1,360 mg/dl) without a direct LDL-C measurement, and one was excluded due to an sLDLR value at 6 months that was judged to be erroneous (more than 10-fold below the baseline level and 3-fold below the next lowest level in the study population). Hence, the present study includes results for 461 participants, 225 randomized to the LF arm and 236 to the HF arm (supplemental Fig. S1).

### Laboratory measurements

Fasting plasma triglycerides, total cholesterol, HDL-C, glucose, insulin, and homeostatic model of insulin resistance (HOMA-IR). were determined as described previously (9). Apolipoproteins B and A-I (apoA-I) were analyzed using K-assay reagents from Kamiya Biomedical Company (Seattle, WA) on a Liasys 330 analyzer (AMS Diagnostics). Lipoprotein particle concentrations and LDL peak diameter were measured by ion mobility, as previously described (10, 11). The particle size intervals for the lipoprotein fractions analyzed here are shown in supplemental Table S1.

Plasma sLDLR concentration was analyzed as part of a panel by Olink Proteomics AB (12, 13) This procedure uses target-specific antibody pairs linked to DNA strands that upon binding to the target analyte create a real-time PCR amplicon in a proximity-dependent manner enabled by the action of a DNA polymerase. The recorded Ct values are converted to a linear scale from a log2 scale providing a measure of analyte concentration as the number of amplicons (13).

### Statistical procedures

Comparisons were made at baseline between this subset and the full DIETFITS population and between the two diet groups in this subset of the DIETFITS study. A two-sample *t*-test was used for continuous measures and Pearson’s Chi-square test for categorical variables. Linear regression was employed to assess baseline associations and correlations between changes from baseline to 6 months, the latter calculated by subtracting the baseline value from the 6 month value.

Natural logarithm transformations were made of measures with skewed distributions prior to regression analyses: triglyceride, large VLDL, small and very small LDL, and insulin. Bonferroni adjustments were made to *P* values to account for multiple testing.

Because of substantial multicollinearity of change values for the lipoprotein fractions, principal component (PC) analysis was used to construct independent linear combinations of these variables that explained the observed variance. PC scores were predicted for each individual and tested for correlations with changes in other clinically significant measures using linear regression. The scores were calculated as a sum of products across lipoprotein fractions, where each product was the PC loading factor for a fraction multiplied by an individual’s change value for the same fraction. These predictions were performed for a limited number of PCs that accounted for the majority of the total variance. All analyses were conducted in Stata (version 15.1, StataCorp LLC).

## Results

Demographic and baseline laboratory data did not differ significantly between the two randomized dietary arms and were not significantly different from those in the full study population (supplemental Table S2).

**Fig. 1:**
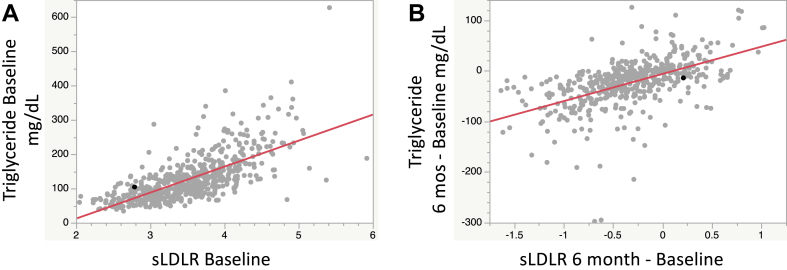
Correlations of baseline and 6 month change values for sLDLR with corresponding values for plasma triglyceride (both significant at *P* < 0.0001). sLDLR values on *x*-axis are in linearized Ct units as described in Materials and Methods section.

Regression models for baseline measurements adjusting for diet group, age, and sex showed significant positive associations of sLDLR with all lipid, apoprotein, and lipoprotein particle concentrations except for LDL-C, HDL-C, apoA1, and large HDL, and an inverse relationship with LDL peak diameter, with the strongest positive relationship found for triglycerides (Table 1 and Fig. 1A). There were also significant positive correlations of sLDLR with glucose, insulin, and HOMA-IR. None of these relationships were altered by further adjustment for BMI (Table 1). Similarly, significance was retained for most correlations after further adjustment for triglyceride, although the strengths of the lipoprotein particle associations were substantially attenuated (supplemental Table S3). Analysis of changes in variables between baseline and 6 months (Table 2 and supplemental Table S4) showed that in comparison with the LF diet, the LC diet resulted in a significantly greater decrease in sLDLR and smaller reductions in total cholesterol and total LDL particles. In addition, compared with the LF diet, the LC diet resulted in increases versus decreases in LDL-C, large LDL, HDL-C, apoA1, and large HDL and a greater increase in LDL peak diameter. There were no sex differences in 6 month changes in the combined diet groups except for a smaller triglyceride decrease for women (*P* = 3.0e-05). Consistent with a recent report (14), weight loss after the first 6 months, adjusted for age and sex, was modestly greater in the LC than LF arms (BMI difference = −0.451, *P* = 0.015) (Table 2). However, the diet response differences were not substantially modified after further adjustment for BMI change (Table 2).

**Table 1:** Baseline associations with sLDLR

Variable	Adjusted for diet, age, and sex			Adjusted for diet, age, sex, and BMI		
	Beta (CI)	*P*	R2	Beta (CI)	*P*	R2
BMI, kg/m^2^	0.114 (0.0593, 0.170)	5.30E-05^a^	0.04	—	—	—
Lipids and apoproteins, mg/dl						
Cholesterol	2.24 (1.72, 2.76)	3.00E-16^a^	0.18	2.31 (1.78, 2.84)	1.2e-16^a^	0.18
Triglycerides	0.0555 (0.0503, 0.0607)	3.50E-68^a^	0.53	0.056 (0.0506, 0.0613)	3.3e-67^a^	0.53
LDL-C	0.698 (0.232, 1.16)	3.40E-03	0.06	0.721 (0.245, 1.20)	3.0e-03	0.06
HDL-C	0.0239 (−0.118, 0.165)	7.40E-01	0.13	0.0485 (−0.0952, 0.192)	5.1e-01	0.14
ApoB	1.82 (1.47, 2.18)	2.30E-21^a^	0.25	1.84 (1.47, 2.20)	6.5e-21^a^	0.25
ApoA1	0.174 (−0.210, 0.557)	3.70E-01	0.17	0.243 (−0.146, 0.633)	2.2e-01	0.18
Lipoprotein particles, nmol/l						
Total VLDL	6.39 (5.46, 7.32)	3.90E-35^a^	0.33	6.6 (5.65, 7.54)	3.6e-36^a^	0.34
Large VLDL	0.0641 (0.0559, 0.0723)	2.50E-43^a^	0.42	0.0657 (0.0574, 0.074)	5.7e-44^a^	0.42
Medium VLDL	3.19 (2.76, 3.61)	1.80E-40^a^	0.38	3.28 (2.85, 3.71)	1.3e-41^a^	0.39
Small VLDL	1.50 (1.10, 1.90)	6.50E-13^a^	0.12	1.56 (1.15, 1.96)	2.3e-13^a^	0.13
IDL	4.22 (3.39, 5.04)	1.50E-21^a^	0.21	4.30 (3.45, 5.14)	1.4e-21^a^	0.22
Total LDL	21.6 (14.9, 28.2)	5.20E-10^a^	0.12	22.5 (15.7, 29.3)	1.8e-10^a^	0.13
Large LDL	−7.94 (−12.3, −3.55)	4.10E-04^a^	0.05	−7.94 (−12.4, −3.47)	5.3e-04^a^	0.05
Medium LDL	5.92 (4.29, 7.54)	3.10E-12^a^	0.17	6.1 (4.45, 7.75)	1.6e-12^a^	0.17
Small LDL	0.0511 (0.043, 0.0593)	2.10E-30^a^	0.33	0.053 (0.0447, 0.0612)	1.5e-31^a^	0.34
Very small LDL	0.0315 (0.0265, 0.0365)	1.10E-30^a^	0.31	0.0324 (0.0274, 0.0375)	2.1e-31^a^	0.32
Total HDL	0.186 (0.100, 0.273)	2.60E-05^a^	0.06	0.194 (0.106, 0.282)	1.7e-05^a^	0.06
Large HDL	0.0167 (−0.0135, 0.0469)	2.80E-01	0.09	0.0189 (−0.0118, 0.0497)	2.3e-01^a^	0.09
Small HDL	0.17 (0.109, 0.231)	6.90E-08^a^	0.06	0.175 (0.113, 0.237)	4.6e-08^a^	0.07
LDL peak diameter, Å	−0.58 (−0.664, −0.496)	2.40E-35^a^	0.38	−0.593 (−0.679, −0.507)	1.1e-35^a^	0.38
Fasting glucose, mg/dl	0.225 (0.0794, 0.370)	2.50E-03	0.11	0.188 (0.0416, 0.335)	1.2e-02	0.12
Fasting insulin, μU/ml	0.0313 (0.0239, 0.0387)	1.20E-15^a^	0.18	0.0263 (0.0192, 0.0335)	2.0e-12^a^	0.27
HOMA-IR	0.0335 (0.0256, 0.0414)	8.40E-16^a^	0.19	0.0282 (0.0206, 0.0358)	1.4e-12^a^	0.28

a Statistically significant at the Bonferroni-adjusted threshold of *P* < 0.002.

**Table 2:** Associations of the LC diet versus the LF diet with changes in BMI and laboratory measurements from baseline to 6 months

Variable	Adjusted for age and sex			Adjusted for age, sex, and BMI		
	Beta (CI) LC versus LF	*P*	R2	Beta (CI) LC versus LF diet	*P*	R2
sLDLR^b^	−1.62 (−2.36, −0.878)	2.10E-05^a^	0.05	−1.25 (−1.93, −0.566)	3.6e-04^a^	0.21
BMI, kg/m^2^	−0.451 (−0.814, −0.0887)	1.50E-02	0.03	—	—	—
Lipids and apoproteins, mg/dl						
Cholesterol	9.20 (4.93, 13.5)	2.80E-05^a^	0.04	9.81 (5.54, 14.1)	8.3e-06^a^	0.05
Triglycerides	−13.7 (−22.0, −5.55)	1.10E-03^a^	0.06	−11.1 (−19.1, −3.1)	6.6e-03	0.12
LDL-C	8.96 (5.26, 12.7)	2.60E-06^a^	0.05	9.04 (5.31, 12.8)	2.5e-06^a^	0.05
HDL-C	2.99 (1.87, 4.11)	2.50E-07^a^	0.06	2.99 (1.86, 4.12)	2.9e-07^a^	0.06
ApoB	2.53 (0.0067, 5.05)	4.90E-02	0.02	3.09 (0.595, 5.59)	1.5e-02	0.05
ApoA1	7.76 (4.83, 10.7)	3.10E-07^a^	0.07	7.86 (4.90, 10.8)	2.7e-07^a^	0.07
Lipoprotein particles, nmol/l						
Total VLDL	1.79 (−7.64, 11.2)	7.10E-01	0.01	3.88 (−5.47, 13.2)	4.1e-01	0.04
Large VLDL	−1.35 (−3.52, 0.827)	2.20E-01	0.02	−0.696 (−2.82, 1.43)	5.2e-01	0.08
Medium VLDL	−1.55 (−5.94, 2.84)	4.90E-01	0.01	−0.539 (−4.89, 3.81)	8.1e-01	0.04
Small VLDL	4.68 (0.646, 8.72)	2.30E-02	0.02	5.12 (1.07, 9.17)	1.3e-02	0.02
IDL	6.39 (−1.39, 14.2)	1.10E-01	0.01	7.86 (0.122, 15.6)	4.7e-02	0.03
Total LDL	96.8 (40.2, 153)	8.50E-04^a^	0.03	110 (53.5, 166)	1.4e-04^a^	0.06
Large LDL	111 (71.5, 151)	6.10E-08^a^	0.07	115 (74.9, 155)	2.7e-08^a^	0.08
Medium LDL	0.58 (−14.1, 15.3)	9.40E-01	0.01	2.85 (−11.9, 17.6)	7.0e-01	0.03
Small LDL	−11.5 (−27.5, 4.57)	1.60E-01	0.02	−8.99 (−25.0, 7.04)	2.7e-01	0.04
Very small LDL	−3.46 (−23.4, 16.5)	7.30E-01	0.02	0.950 (−18.8, 20.7)	9.2e-01	0.05
Total HDL	1.11 (0.363, 1.85)	3.60E-03	0.02	1.25 (0.509, 1.99)	9.9e-04^a^	0.04
Large HDL	0.544 (0.313, 0.774)	4.80E-06^a^	0.05	0.546 (0.314, 0.779)	5.0e-06^a^	0.05
Small HDL	0.563 (0.0089, 1.12)	4.60E-02	0.01	0.703 (0.156, 1.25)	1.2e-02	0.05
LDL peak diameter, Å	1.86 (1.13, 2.60)	9.90E-07^a^	0.09	1.70 (0.968, 2.43)	6.5e-06^a^	0.12
Fasting glucose, mg/dl	1.03 (−0.695, 2.75)	2.40E-01	0.01	1.42 (−0.288, 3.13)	1.0e-01	0.04
Fasting insulin, μU/ml	−0.403 (−2.53, 1.73)	7.10E-01	0.01	0.008 (−2.11, 2.13)	9.9e-01	0.04
HOMA-IR	−0.0385 (−0.587, 0.510)	8.90E-01	0.01	0.0743 (−0.470, 0.619)	7.9e-01	0.04

a Statistically significant at the Bonferroni-adjusted threshold of *P* < 0.002.

b Measured as linearized Ct units as described in the Materials and Methods section.

The relationships of BMI change with changes in sLDLR and plasma lipids, apolipoproteins, and lipoproteins are shown in Table 3. BMI change was significantly positively associated with changes in sLDLR, triglycerides, apoB, total, large, and medium VLDL, IDL, total and very small LDL, and small HDL, and inversely with LDL peak diameter, and these associations were independent of diet assignment (supplemental Table S5). Notably, none of the associations between changes in BMI and the lipid, apoprotein, and lipoprotein measures remained significant after adjustment for sLDLR, with the exception of small HDL, although a positive association with large LDL change became significant (Table 3). Changes in BMI were positively associated with each of the glycemic measures independent of diet (Table 3 and supplemental Table S5), and the associations with fasting insulin and HOMA-IR change were slightly weakened and no longer significant after adjustment for sLDLR (Table 3).

**Table 3:** Associations of change in BMI with changes in laboratory measurements from baseline to 6 months

Changes in	Adjusted for age and sex			Adjusted for age, sex, and sLDLR		
	Beta (CI)	*P*	R2	Beta (CI)	*P*	R2
sLDLR^b^	0.852 (0.679, 1.03)	2.80E-20^a^	—	—	—	—
Lipids and apoproteins, mg/dl						
Cholesterol	1.08 (−0.0076, 2.18)	5.20E-02	0.01	−0.0562 (−1.23, 1.12)	9.3e-01	0.06
Triglycerides	6.23 (4.22, 8.24)	2.40E-09^a^	0.11	0.988 (−0.886, 2.86)	3.0e-01	0.36
LDL-C	−0.0807 (−1.03, 0.873)	8.70E-01	0.01	−0.127 (−1.18, 0.921)	8.1e-01	0.01
HDL-C	−0.0794 (−0.370, 0.211)	5.90E-01	0	−0.126 (−0.445, 0.193)	4.4e-01	0.00
ApoB	1.16 (0.536, 1.79)	3.00E-04^a^	0.04	0.339 (−0.327, 1.01)	3.2e-01	0.11
ApoA1	−0.0068 (−0.768, 0.754)	9.90E-01^a^	0.02	−0.255 (−1.09, 0.579)	5.5e-01	0.02
Lipoprotein particles, nmol/l						
Total VLDL	4.53 (2.19, 6.87)	1.60E-04^a^	0.04	1.73 (−0.767, 4.22)	1.7e-01	0.09
Large VLDL	1.46 (0.929, 1.99)	1.10E-07^a^	0.08	0.458 (−0.0814, 0.998)	9.6e-02	0.21
Medium VLDL	2.25 (1.17, 3.34)	5.50E-05^a^	0.04	0.715 (−0.429, 1.86)	2.2e-01	0.12
Small VLDL	0.819 (−0.201, 1.84)	1.20E-01	0.01	0.552 (−0.567, 1.67)	3.3e-01	0.01
IDL	3.04 (1.1, 4.99)	2.20E-03	0.03	1.95 (−0.174, 4.07)	7.2e-02	0.04
Total LDL	25.2 (10.9, 39.5)	5.60E-04^a^	0.03	23.9 (8.22, 39.6)	2.9e-03	0.03
Large LDL	4.76 (−5.54, 15.1)	3.60E-01	0.01	18 (7.04, 28.9)	1.3e-03^a^	0.08
Medium LDL	4.95 (1.27, 8.62)	8.50E-03	0.03	3.59 (−0.443, 7.61)	8.1e-02	0.03
Small LDL	5.74 (1.73, 9.75)	5.10E-03	0.03	−0.308 (−4.5, 3.89)	8.9e-01	0.12
Very small LDL	9.75 (4.80, 14.7)	1.20E-04^a^	0.05	2.64 (−2.56, 7.84)	3.2e-01	0.13
Total HDL	0.281 (0.0937, 0.468)	3.40E-03	0.02	0.27 (0.0639, 0.476)	1.0e-02	0.02
Large HDL	−0.0093 (−0.0687, 0.0502)	7.60E-01	0.01	0.0146 (−0.0505, 0.0797)	6.6e-01	0.01
Small HDL	0.290 (0.153, 0.428)	4.00E-05^a^	0.04	0.255 (0.104, 0.406)	9.7e-04^a^	0.04
LDL peak diameter, Å	−0.413 (−0.600, −0.226)	1.80E-05^a^	0.08	−0.0526 (−0.242, 0.137)	5.8e-01	0.22
Fasting glucose, mg/dl	0.824 (0.396, 1.25)	1.70E-04^a^	0.04	0.944 (0.474, 1.41)	9.1e-05^a^	0.04
Fasting insulin, μU/l	0.910 (0.380, 1.44)	8.00E-04^a^	0.04	0.854 (0.272, 1.44)	4.1e-03^a^	0.04
HOMA-IR	0.248 (0.112, 0.384)	3.80E-04^a^	0.04	0.238 (0.0889, 0.388)	1.8e-03^a^	0.04

a Statistically significant at the Bonferroni-adjusted threshold of *P* < 0.002.

b Measured as linearized Ct units as described in the Materials and Methods section.

Regression analyses, with adjustment for age, sex, BMI change, and diet assignment, were performed to test the relations of 6 month changes in sLDLR with changes in each of the laboratory measurements (Table 4). Changes in sLDLR were significantly positively associated with change in total cholesterol, triglycerides (Fig. 1B), apoB, total, large, and medium VLDL, and small and very small LDL; and inversely with large LDL and peak LDL diameter. Comparison of the separate regressions for the LC and LF diet groups showed no significant differences in sLDLR effects, except for a weak inverse association with HDL-C on the LC diet versus a positive association on the LF diet (supplemental Table S6).

**Table 4:** Associations of change in sLDLR with changes in other laboratory measurements from baseline to 6 months

Changes in	Adjusted for diet, sex, age, and BMI			Adjusted for diet, sex, age, BMI, and triglycerides		
	Beta (CI)	*P*	R2	Beta (CI)	*P*	R2
Lipids and apoproteins, mg/dl						
Cholesterol	1.60 (1.04, 2.16)	3.10E-08^a^	0.12	1.12 (0.477, 1.77)	7.1e-04^a^	0.13
Triglycerides	6.07 (5.15, 6.99)	5.60E-33^a^	0.36	—	—	—
LDL-C	0.261 (−0.241, 0.763)	3.10E-01	0.06	1.08 (0.514, 1.65)	2.1e-04^a^	0.11
HDL-C	0.124 (−0.0281, 0.276)	1.10E-01	0.06	0.0408 (−0.137, 0.218)	6.5e-01	0.07
ApoB	1.07 (0.744, 1.39)	2.20E-10^a^	0.13	0.771 (0.396, 1.15)	6.2e-05^a^	0.15
ApoA1	0.478 (0.0815, 0.875)	1.80E-02	0.08	0.266 (−0.198, 0.729)	2.6e-01	0.09
Lipoprotein particles, nmol/l						
Total VLDL	3.48 (2.26, 4.70)	3.80E-08^a^	0.10	1.43 (0.0458, 2.81)	4.3e-02	0.16
Large VLDL	1.19 (0.929, 1.46)	1.90E-17^a^	0.21	0.194 (−0.0607, 0.449)	1.4e-01	0.47
Medium VLDL	1.84 (1.28, 2.41)	2.90E-10^a^	0.12	0.655 (0.031, 1.28)	4.0e-02	0.21
Small VLDL	0.439 (−0.107, 0.984)	1.10E-01	0.03	0.580 (−0.0582, 1.22)	7.5e-02	0.03
IDL-1	1.50 (0.466, 2.54)	4.60E-03	0.05	1.79 (0.579, 3.00)	3.9e-03	0.05
Total LDL	4.07 (−3.50, 11.6)	2.90E-01	0.07	0.680 (−8.16, 9.52)	8.8e-01	0.07
Large LDL	−13.3 (−18.6, −8.10)	8.00E-07^a^	0.13	−9.86 (−16.0, −3.76)	1.6e-03^a^	0.14
Medium LDL	1.71 (−0.272, 3.68)	9.10E-02	0.03	1.15 (−1.17, 3.46)	3.3e-01	0.03
Small LDL	7.09 (5.03, 9.15)	4.10E-11^a^	0.12	5.06 (2.67, 7.45)	3.8e-05^a^	0.14
Very small LDL	8.60 (6.05, 11.1)	9.90E-11^a^	0.14	4.34 (1.45, 7.23)	3.4e-03	0.19
Total HDL	0.0419 (−0.0579, 0.142)	4.10E-01	0.04	−0.0367 (−0.153, 0.0795)	5.4e-01	0.06
Large HDL	−0.0164 (−0.0477, 0.015)	3.00E-01	0.05	−0.0283 (−0.065, 0.0084)	1.3e-01	0.06
Small HDL	0.0583 (−0.0153, 0.132)	1.20E-01	0.06	−0.0084 (−0.0938, 0.077)	8.5e-01	0.07
LDL peak diameter, Å	−0.396 (−0.488, −0.305)	3.00E-16^a^	0.24	−0.248 (−0.352, −0.143)	4.0e-06^a^	0.28
Fasting glucose, mg/dl	−0.112 (−0.342, 0.118)	3.40E-01	0.04	−0.229 (−0.498, 0.0397)	9.5e-02	0.05
Fasting insulin, μU/l	0.0677 (−0.218, 0.353)	6.40E-01	0.04	0.0282 (−0.307, 0.363)	8.7e-01	0.04
HOMA-IR	0.0131 (−0.0604, 0.0866)	7.30E-01	0.04	0.0059 (−0.0802, 0.092)	8.9e-01	0.04

a Statistically significant at the Bonferroni-adjusted threshold of *P* < 0.002.

In view of the strong correlations between sLDLR among both baseline levels and changes of the multiple lipoprotein subfractions (supplemental Table S7), PC analysis was performed to identify independent clusters of their change measurements with and without inclusion of sLDLR (Table 5). PCs 1–3 accounted for 78% of the total variance of the lipoprotein subfractions. With inclusion of sLDLR in the PC analysis, the results were minimally changed, and sLDLR was most heavily loaded in PC2. This PC, which accounted for 18% of the total variance, was positively loaded (>0.3) with large VLDL and small and very small LDL and inversely with large LDL and large HDL. As such, PC2 is highly consistent with the atherogenic lipoprotein phenotype (15) that has also been identified in previous studies using PC analysis. PC1, with similar loadings across lipoprotein fractions, and which accounted for the largest proportion of the total variance (47%), is considered to capture the underlying multicollinearity of the majority of the lipoprotein fractions. Finally, PC3, accounting for 13% of the total variance, represented negative weighting of all VLDL fractions and positive weighting of medium and small LDL. Hence, this PC may represent a precursor-product relationship between VLDL and the most abundant LDL particles that does not involve a significant role for sLDLR.

**Table 5:** PC analysis of changes in lipoprotein fractions from baseline to 6 months

Eigenvalues: Portion of analysis explained by linear combinations of variables for each orthogonal dimensions, each represented by one PC									
Eigenvalues, lipoprotein fractions only					Eigenvalues, lipoprotein fractions, and sLDLR				
Component	Eigenvalue	Difference	Proportion	Cumulative variance	Component	Eigenvalue	Difference	Proportion	Cumulative variance
1	4.700	2.883	0.470	0.470	Comp1	4.772	2.634	0.434	0.434
2	1.817	0.562	0.182	0.652	Comp2	2.138	0.870	0.194	0.628
3	1.255	0.407	0.126	0.777	Comp3	1.268	0.411	0.115	0.743

Most relevant eigenvectors: loading of each variable on the three PCs with eigenvalues >1									
Top 3 eigenvectors, lipoprotein fractions only					Top 3 eigenvectors, lipoprotein fractions, and sLDLR				
Variable	Comp1	Comp2	Comp3	Unexplained Variance	Variable	Comp1	Comp2	Comp3	Unexplained Variance
Large VLDL	0.279	0.327	−0.438	0.199	Large VLDL	0.290	0.303	−0.395	0.206
Medium VLDL	0.359	0.183	−0.443	0.086	Medium VLDL	0.363	0.155	−0.409	0.108
Small VLDL	0.368	−0.174	−0.258	0.224	Small VLDL	0.359	−0.182	−0.266	0.223
Small IDL	0.407	−0.103	0.044	0.202	Small IDL	0.400	−0.124	0.035	0.202
Large LDL	0.239	−0.564	0.036	0.151	Large LDL	0.220	−0.520	−0.031	0.189
Medium LDL	0.255	0.048	0.549	0.312	Medium LDL	0.253	−0.001	0.543	0.322
Small LDL	0.241	0.469	0.454	0.070	Small LDL	0.249	0.360	0.512	0.093
Very small LDL	0.252	0.411	0.036	0.393	Very small LDL	0.260	0.326	0.100	0.438
Large HDL	0.319	−0.308	0.103	0.335	Large HDL	0.305	−0.320	0.076	0.330
Small HDL	0.383	−0.121	0.153	0.256	Small HDL	0.375	−0.145	0.140	0.259
					sLDLR	0.139	0.454	−0.113	0.451

PC analysis conducted with z-standardized changes in lipoprotein fractions from baseline to 6 months.

Finally, regression analyses were performed to assess the relationships of changes in the lipoprotein subfraction-based PCs with changes in sLDLR and BMI and with diet assignment (supplemental Table S8). Change in sLDLR was highly significantly associated with PC2, explaining 21.4% of its variance, less strongly with PC1, and not significantly with PC3. Assignment to the LC versus LF diet was significantly associated with change in PC2 but not to the other PCs.

## Discussion

The present findings demonstrate that changes in plasma concentration of sLDLR, the proteolytically cleaved ligand-binding ectodomain of the LDLR, were significantly associated with changes in levels of a group of interrelated lipids and lipoprotein subfractions following a 6 month trial of either a healthy LC diet or LF diet aimed at achieving weight loss. These included positive associations with total cholesterol and triglyceride, apoB, large and medium VLDL, and small and very small LDL, as well as inverse associations with large LDL and peak LDL diameter. PC analysis revealed that these lipid and lipoprotein changes were distributed across three independent PCs comprising 78% of the total variance. PC2 was loaded with changes in the cluster of VLDL and LDL fractions noted above as well as inversely with change in large HDL. The components of PC2 are consistent with those previously shown for a genetically influenced trait associated with increased risk of cardiovascular disease, which thus has been designated atherogenic lipoprotein phenotype (ALP) (15). Several previous studies have also identified PCs consistent with the characteristics of ALP (16, 17, 18, 19). Moreover, the association of PC2 with BMI in the present study, as well as its inverse relationship to assignment to the LC diet versus the LF diet, is consistent with previous findings showing that both reduced adiposity and carbohydrate intake diminish expression of ALP (20, 21). Notably, among the PCs in the present study, change in sLDLR was most strongly associated with change in PC2, explaining one-fifth of its variance.

There is evidence that the primary metabolic factor promoting expression of ALP is an increase in large triglyceride-enriched VLDL particles, because of their increased hepatic secretion and/or reduced clearance, and that this promotes a shift in the predominant LDL species from larger to smaller particles resulting in a decrease in peak LDL diameter, as well as a reduction of larger HDL particles (22). Notably, among the components of PC2, the strongest associations with sLDLR, both for baseline and change values, were observed for large VLDL as well as plasma triglyceride, suggesting that these metabolic drivers of ALP also affect the generation and/or clearance of sLDLR in plasma.

The present findings are consistent with previous studies showing cross-sectional associations of sLDLR with plasma triglyceride concentration (4, 5, 6), and a report in a study of children with familial hypercholesterolemia that sLDLR is also correlated with small LDL (5). We here show for the first time that these relationships, and those involving other components of ALP, are also observed for changes over time in the context of a dietary weight loss trial. While the basis for these correlations has not been established, it has been reported, based on gel permeation chromatography and immunoprecipitation, that sLDLR can be coisolated with VLDL from human plasma, with much smaller amounts isolated with LDL and HDL (3). It is possible that such a physical association is responsible at least in part for the preferential correlations of sLDLR with plasma triglyceride and triglyceride-rich VLDL particles, though it also has been suggested that sLDLR-lipoprotein complexes may compete with native lipoproteins for LDLR-mediated plasma clearance (3).

The production of sLDLR has been shown to be mediated by proteolysis of cell surface LDLR by MT1-MMP, with genetic knockdown or overexpression of MT1-MMP in cellular and mouse models leading to reciprocal changes in hepatocellular LDLR content (3). Consistent with these effects, plasma cholesterol levels were reduced in mice with liver-specific MT1-MMP knockdown, and increased with its overexpression (3), with parallel effects on aortic atherogenesis (3). However, as in the present study, correlations of plasma LDL-C levels with sLDLR in humans have generally been much weaker than those for triglyceride (4, 5, 6). This may reflect our finding that sLDLR is correlated with small cholesterol-depleted LDL particles, which have been shown to have relatively low affinity for LDLR (23), and which are derived, as noted above, from a pathway related to metabolism of large triglyceride-enriched VLDL. Thus, it is possible that the sLDLR-lipoprotein associations observed here result primarily from a change in conformation of the proteolytically cleaved LDLR ectodomain that preserves or increases its affinity for VLDL particles. However, it has not been established whether sLDLR directly promotes increased plasma VLDL particle concentrations, for example by reducing their clearance, or whether the correlations of sLDLR with VLDL and other lipoproteins reflect its attachment to them in plasma following cellular shedding. With regard to the latter possibility, since as noted above both weight loss and reduced carbohydrate intake are known to decrease expression of ALP, it may be that the changes in sLDLR resulting from the dietary interventions in the present study primarily reflect its physical association in plasma with particles in the VLDL metabolic pathway.

While as noted above, the present study did not show a strong relationship between sLDLR and plasma LDL-C, we have observed significant correlations of sLDLR with plasma proprotein convertase subtilisin/kexin type 9 (PCSK9), a protein that promotes cellular LDLR degradation (23), for both baseline (*P* = 8.6e-33) and for 6 month change values (*P* = 2.6e-11), adjusted for age, sex, and. diet (Krauss R.M., personal communication). Since hepatic expression of sLDLR and PCSK9 is coregulated by SREBP2, this suggests that reduced hepatocellular cholesterol because of MT1-MMP-mediated LDLR proteolysis may have led to upregulation of SREBP2, resulting in increased expression of both genes, and correlations of the levels of sLDLR and PCSK9 in plasma. However, PCSK9 neither is responsible for generating sLDLR (24) nor is it a substrate for MT1-MMP (3) and thus, the lipoprotein associations with sLDLR shown here are not mediated by changes in PCSK9. Interestingly, it has recently been reported that cell surface LDLR shedding is increased with PCSK9 deficiency in female mice but not male mice and in women but not men treated with a PCSK9 monoclonal antibody (25). However, we found no interaction by sex for the association between plasma PCSK9 and sLDLR, both for baseline and change values, suggesting that such a female sex-dependent inverse relationship between cellular PCSK9 and LDLR shedding was not a factor in the present study.

MT1-MMP is responsible for cleavage and shedding of multiple cell surface proteins other than LDLR (26), including the insulin receptor (27). It has been reported in mouse models that insulin sensitivity is impaired by MT1-MMP overexpression in conjunction with increased production of the soluble insulin receptor (sIR) ectodomain, and the opposite effects are induced by MT1-MMP inhibition (27). Notably, plasma levels of sIR have been found to be increased in patients with diabetes and to correlate with glucose concentrations (28), consistent with a role for MT1-MMP activity in modulating insulin sensitivity. The key role of insulin resistance in the atherogenic dyslipidemia of the metabolic syndrome (29) raises the possibility that concordant changes in MT1-MMP-mediated generation of sIR and sLDLR might have contributed to the relationships observed between sLDLR and the lipoprotein components of this dyslipidemia. However, after adjustment for change in BMI, there was no significant relationship between change in sLDLR and insulin sensitivity as assessed by HOMA-IR, suggesting that sIR is not responsible for the lipid and lipoprotein associations with sLDR observed here.

An unexpected finding in the present study was that none of the observed associations between changes in BMI and lipid, apoprotein, and lipoprotein measures except for small HDL particles remained significant after adjustment for sLDLR. While as noted above, a reduction in features of ALP with weight loss has been well documented (20, 21) it may be that a stronger association of change in ALP with sLDLR than with BMI change accounts for the present finding.

There are several limitations to the present study. While it was designed to recruit a diverse study cohort, its results may not be generalizable, and in this regard, replication in an independent study population would be desirable. It is also possible that the likelihood of complete follow-up at 6 months may have been related to a baseline characteristic, diet assignment, or weight loss, introducing potential bias in the estimates shown here. In addition, relationships observed between measurements at two time points may fail to capture biologically meaningful effects, such as those affecting body weight, that occur at different rates within this interval or thereafter. Nevertheless, several of the observed associations are consistent with those reported in other studies, supporting their validity. Finally, since dietary intake was not controlled in DIETFITS, and compliance to the prescribed diets may have changed over time, the role of diet in mediating changes in plasma sLDLR and lipoprotein levels in this study cannot be determined conclusively.

In conclusion, the present results have shown that the circulating level of plasma sLDLR is a component of ALP that can, along with ALP, be reduced by weight loss and limitation of carbohydrate intake. While a causal role for cellular LDLR shedding in cardiovascular disease has not been established, it remains possible that decreased MT1-MMP-mediated LDLR proteolysis signifies a process that can favorably impact atherogenesis in part by reducing levels of ALP components. This possibility, taken together with the reciprocity between sLDLR and cellular LDLR content, as well as the role of MT1-MMP in atherogenesis, would support the suggestion of MT1-MMP inhibition as a potential target of therapy aimed at improving atherogenic dyslipidemia and lowering CVD risk (3).

## Data availability

Can be provided upon request.

## Supplemental data

This article contains supplemental data.

## Conflict of interest

R. M. K., research funding from 10.13039/100015627Quest Diagnostics, royalties from patent on ion mobility methodology for lipoprotein analysis. All other authors declare that they have no conflicts of interest with the contents of this article.
