# Supplementary material for: Changes in soluble LDL receptor and lipoprotein fractions in response to diet in the DIETFITS weight loss study
Source: J Lipid Res. 2024 Jan 19;65(3):100503. doi: 10.1016/j.jlr.2024.100503 (PMC10882123; doi:10.1016/j.jlr.2024.100503)
Supplement: Supplementary Figures [file mmc1.docx]

**SUPPLEMENTARY FIGURE**

**Figure S1. Flow of Participants**

632 Randomized

314 Low-fat diet

318 Low-carbohydrate diet

9 withdrew prior to receiving diet assignment

14 withdrew prior to receiving diet assignment

304 Informed of diet assignment

305 Informed of diet assignment

BASELINE

300 with lipid particle concentrations

304 with sLDLR concentrations

304 with glycemic measures

BASELINE

304 with lipid particle concentrations

305 with sLDLR concentrations

305 with glycemic measures

20 withdrew after BL visit

27 withdrew after 3M visit

24 withdrew after BL visit

13 withdrew after 3M visit

258 completed 6M physical visit

267 completed 6M physical visit

6 MONTHS

225 with lipid particle concentrations

225 with sLDLR concentrations

225 with glycemic measures

6 MONTHS

239 with lipid particle concentrations

239 with sLDLR concentrations

239 with glycemic measures

1 subject dropped due to failure in laboratory assessments

2 subjects dropped due to failures in laboratory assessments

224 with complete data for analysis of baseline to 6-month changes

237 with complete data for analysis of baseline to 6-month changes
