# Supplementary material for: Changes in soluble LDL receptor and lipoprotein fractions in response to diet in the DIETFITS weight loss study
Source: J Lipid Res. 2024 Jan 19;65(3):100503. doi: 10.1016/j.jlr.2024.100503 (PMC10882123; doi:10.1016/j.jlr.2024.100503)
Supplement: Supplementary Tables [file mmc2.docx]

**SUPPLEMENTARY TABLES**

**Table S1. Lipoprotein fraction size intervals determined by ion mobility analysis**

| **Lipoprotein Fractions** | **Particle Size (nm)** |
| --- | --- |
| **VLDL** |  |
| Large | 42.4 - 52 |
| Medium | 33.5 - 42.4 |
| Small | 29.6 - 33.5 |
| **IDL** | 25 - 29.6 |
| **LDL** |  |
| Large | 22- 25 |
| Medium | 21.41 - 22 |
| Small | 20.82 - 21.41 |
| Very Small | 18 - 20.82 |
| **HDL** |  |
| Large | 10.5 - 14.5 |
| Small | 7.65 - 10.5 |

**Table S2A. Comparison of the full study population at baseline with the subset studied here**

|  | ***All Subjects*** | | | ***LC Diet Group*** | | | ***LF Diet Group*** | | |
| --- | --- | --- | --- | --- | --- | --- | --- | --- | --- |
|  | **Original Study (n)** | **Subset (n)** | **P**  **value** | **Original Study (n)** | **Subset (n)** | **P value** | **Original Study (n)** | **Subset (n)** | **P value** |
| **Diet** | 609 | 461 | .63 | - | - | - | - | - | - |
| Low Carbohydrate | 304 | 237 |  |  | - | - | - | - |  |
| Low Fat | 305 | 224 |  |  | - | - | - | - |  |
| **Sex** | 609 | 461 | .48 | 304 | 237 | .73 | 305 | 224 | .52 |
| Female | 346 | 272 |  | 179 | 143 |  | 167 | 129 |  |
| Male | 263 | 189 |  | 125 | 94 |  | 138 | 95 |  |
| **Race/Ethnicity** | 606 | 460 | .98 | 303 | 237 | .99 | 303 | 223 | .998 |
| White | 358 | 278 |  | 182 | 145 |  | 176 | 133 |  |
| Hispanic | 128 | 92 |  | 61 | 45 |  | 67 | 47 |  |
| Asian | 61 | 49 |  | 31 | 27 |  | 30 | 22 |  |
| African American | 23 | 14 |  | 13 | 8 |  | 10 | 6 |  |
| Am Ind/Alaskan/Pac Islander | 6 | 4 |  | 3 | 2 |  | 3 | 2 |  |
| Other | 30 | 23 |  | 13 | 10 |  | 17 | 13 |  |

**Table S2B. Comparison of the full study population at baseline with the subset studied here.**

|  | ***Original Study (n=609)*** | | | ***Subset (n=461)*** | | | ***P-values Overall & By Diet Subgroup*** | | |
| --- | --- | --- | --- | --- | --- | --- | --- | --- | --- |
|  | **Mean (SD)** | **Mean LC (SD)** | **Mean LF (SD)** | **Mean (SD)** | **Mean LC (SD)** | **Mean LF (SD)** | **Overall** | **LC** | **LF** |
| Age (yrs) | 39.3 (6.77) | 39.7 (6.73) | 38.9 (6.79) | 39.8 (6.65) | 40.4 (6.65) | 39.3 (6.63) | .183 | .263 | .475 |
| BMI Kg/M^2^ | 33.4 (3.35) | 33.3 (3.4) | 33.4 (3.3) | 33.3 (3.36) | 33.1 (3.39) | 33.4 (3.32) | .619 | .495 | .988 |
| sLDLR* | 12.5 (5.97) | 12.4 (6.04) | 12.6 (5.9) | 12.5 (5.57) | 12.2 (5.22) | 12.8 (5.91) | .894 | .750 | .609 |
| ***Lipids & Apoproteins mg/dL*** |  |  |  |  |  |  |  |  |  |
| Cholesterol | 188 (33.6) | 189 (31.9) | 187 (35.2) | 189 (34.2) | 191 (32.2) | 188 (36.1) | .477 | .449 | .804 |
| Triglycerides | 128 (81.8) | 128 (91.4) | 129 (71.2) | 127 (62.6) | 126 (59.6) | 129 (65.7) | .842 | .710 | .898 |
| LDL-C | 113 (28.4) | 114 (26.1) | 112 (30.5) | 114 (28.7) | 115 (26.1) | 112 (31.2) | .588 | .533 | .882 |
| HDL-C | 49.6 (8.93) | 49.9 (8.98) | 49.4 (8.89) | 50 (9.05) | 50.3 (8.84) | 49.6 (9.27) | .557 | .595 | .782 |
| Apo B | 95.1 (24.4) | 95.3 (23.3) | 94.9 (25.5) | 96.1 (24.8) | 96.6 (23.2) | 95.5 (26.4) | .505 | .498 | .784 |
| Apo A1 | 146 (24.1) | 147 (23.7) | 145 (24.6) | 147 (25.1) | 148 (24.2) | 146 (25.9) | .402 | .460 | .671 |
| ***Lipoprotein Particles nmol/L*** |  |  |  |  |  |  |  |  |  |
| Total VLDL | 174 (67.9) | 172 (63.7) | 176 (71.9) | 175 (68) | 174 (64.9) | 176 (71.2) | .888 | .854 | .969 |
| Large VLDL | 26.8 (16.7) | 26 (14.6) | 27.7 (18.5) | 26.8 (16.5) | 26.0 (15.0) | 27.7 (17.9) | .983 | .952 | .989 |
| Medium VLDL | 71.6 (32.2) | 70.9 (29.8) | 72.3 (34.5) | 71.8 (32.2) | 71.3 (30.3) | 72.4 (34.2) | .918 | .878 | .987 |
| Small VLDL | 75.6 (25.1) | 75.6 (24.9) | 75.7 (25.3) | 76 (25.4) | 76.2 (25.3) | 75.9 (25.5) | .814 | .800 | .939 |
| IDL | 173 (55.6) | 173 (56.2) | 173 (55) | 174 (55.5) | 173 (56.3) | 175 (54.8) | .768 | .871 | .794 |
| Total LDL | 1541 (435) | 1540 (418) | 1542 (451) | 1539 (425) | 1542 (417) | 1537 (434) | .941 | .960 | .880 |
| Large LDL | 795 (272) | 799 (261) | 791 (282) | 795 (268) | 804 (262) | 785 (275) | .994 | .818 | .804 |
| Medium LDL | 234 (106) | 234 (106) | 234 (106) | 234 (106) | 232 (106) | 235 (107) | .912 | .833 | .952 |
| Small LDL | 191 (124) | 191 (124) | 191 (125) | 192 (125) | 190 (125) | 193 (125) | .942 | .940 | .853 |
| Very Small LDL | 321 (150) | 316 (141) | 325 (158) | 319 (143) | 315 (142) | 323 (144) | .853 | .958 | .855 |
| Total HDL | 25.5 (5.27) | 25.5 (5.14) | 25.6 (5.41) | 25.5 (5.30) | 25.5 (5.12) | 25.6 (5.48) | .956 | .982 | .915 |
| Large HDL | 7.08 (1.87) | 7.05 (1.74) | 7.11 (1.98) | 7.07 (1.89) | 7.01 (1.72) | 7.12 (2.06) | .889 | .803 | .963 |
| Small HDL | 18.4 (3.78) | 18.4 (3.77) | 18.5 (3.8) | 18.5 (3.75) | 18.4 (3.72) | 18.5 (3.79) | .883 | .933 | .897 |
| LDL Peak Diameter Å | 221 (6.35) | 221 (6.39) | 221 (6.3) | 221 (6.36) | 221 (6.35) | 221 (6.36) | .962 | .869 | .897 |
| Fasting Glucose mg/dL | 98.5 (9.17) | 98.5 (9.72) | 98.6 (8.6) | 98.4 (9.17) | 98.4 (10.0) | 98.4 (8.22) | .846 | .982 | .787 |
| Fasting Insulin µU/L | 15.7 (11.1) | 15.5 (8.02) | 15.9 (13.5) | 15.3 (11.8) | 14.9 (7.72) | 15.7 (14.9) | .643 | .450 | .925 |
| HOMA-IR | 3.87 (2.92) | 3.82 (2.13) | 3.92 (3.54) | 3.78 (3.08) | 3.69 (2.05) | 3.88 (3.88) | .636 | .479 | .898 |

LC:low-carbohydrate diet; LF: low-fat diet. * Measured as linearized CT units as described in Methods

**Table S3. Baseline associations with sLDLR adjusted for diet, age, sex, BMI and triglycerides**

|  | **beta (CI)** | **p-value** | **R2** |
| --- | --- | --- | --- |
| ***Lipids and Apoproteins mg/dL*** |  |  |  |
| Cholesterol | 1.29 (.566, 2.01) | 5.0e-04 * | 0.21 |
| LDL-C | .877 (.216, 1.54) | 9.4e-03 | 0.06 |
| HDL-C | .273 (.0752, .471) | 6.9e-03 | 0.15 |
| Apo B | .591 (.108, 1.07) | 1.6e-02 | 0.33 |
| Apo A1 | .780 (.243, 1.32) | 4.5e-03 | 0.19 |
| ***Lipoprotein Particles nmol/L*** |  |  |  |
| Total VLDL | 2.48 (1.28, 3.67) | 5.4e-05 | 0.45 |
| Large VLDL | .0052 (-.0031, .0136) | 2.2e-01 | 0.70 |
| Medium VLDL | 1.01 (.492, 1.53) | 1.4e-04 | 0.54 |
| Small VLDL | 1.13 (.572, 1.69) | 8.4e-05 | 0.14 |
| IDL | 3.19 (2.03, 4.35) | 1.1e-07 | 0.23 |
| Total LDL | 5.06 (-4.1, 14.2) | 2.8e-01 | 0.18 |
| Large LDL | -5.53 (-11.7, .685) | 8.1e-02 | 0.05 |
| Medium LDL | .209 (-1.95, 2.37) | 8.5e-01 | 0.27 |
| Small LDL | .0136 (.0034, .0239) | 9.0e-03 | 0.47 |
| Very Small LDL | .0149 (.0082, .0215) | 1.4e-05 | 0.39 |
| Total HDL | .116 (-.0059, .238) | 6.2e-02 | 0.07 |
| Large HDL | .0501 (.0074, .0927) | 2.1e-02 | 0.10 |
| Small HDL | .0658 (-.0192, .151) | 1.3e-01 | 0.09 |
| LDL Peak Diameter Å | -.124 (-.226,-.0228) | 1.7e-02 | 0.55 |
| Fasting Glucose mg/dL | -.0196 (-.222, .183) | 8.5e-01 | 0.14 |
| Fasting Insulin µU/L | .0069 (-.0027, .0165) | 1.6e-01 | 0.32 |
| HOMA-IR | .0066 (-.0036, .0168) | 2.0e-01 | 0.33 |
| All data are for the subgroup of participants studied at baseline and 6 months  * Statistically significant at the Bonferroni-adjusted threshold of P<.002 | | | |

**Table S4. Differences in BMI and laboratory measurements between baseline and 6 months**

|  | Mean BL | Mean 6M | Diff | p-value | Mean BL LC | Mean 6M LC | Diff LC | p-value LC | Mean BL LF | Mean 6M LF | Diff LF | p-value LF | p-value LC vs LF |
| --- | --- | --- | --- | --- | --- | --- | --- | --- | --- | --- | --- | --- | --- |
| BMI Kg/M^2^ | 33.26 | 30.75 | -2.51 | 2.7e-26 * | 33.09 | 30.35 | -2.74 | 1.8e-16 * | 33.44 | 31.18 | -2.26 | 1.3e-11 * | 9.0e-03 n |
| ***Lipids and Apoproteins mg/dL*** |  |  |  |  |  |  |  |  |  |  |  |  |  |
| Cholesterol | 189.2 | 184.1 | -5.10 | 2.6e-02n | 190.7 | 190.2 | -0.53 | 8.6e-01 n | 187.6 | 177.6 | -9.92 | 3.6e-03 n | 1.8e-05 * |
| Triglycerides | 127.4 | 104.6 | -22.83 | 3.7e-09 * | 125.6 | 96.11 | -29.49 | 1.2e-08 * | 129.4 | 113.6 | -15.78 | 6.1e-03 n | 1.3e-03 n |
| LDL Cholesterol | 113.7 | 113.6 | -0.16 | 9.3e-01n | 115.3 | 119.6 | 4.27 | 9.0e-02 n | 112.1 | 107.2 | -4.86 | 9.1e-02 n | 1.6e-06 * |
| Apo B | 96.08 | 91.79 | -4.29 | 7.9e-03n | 96.62 | 93.56 | -3.06 | 1.5e-01 n | 95.52 | 89.92 | -5.60 | 2.3e-02 n | 4.8e-02 n |
| HDL Cholesterol | 49.96 | 49.59 | -0.36 | 5.4e-01n | 50.28 | 51.37 | 1.09 | 1.7e-01 n | 49.62 | 47.71 | -1.91 | 2.6e-02 n | 1.9e-07 * |
| Apo A1 | 147.2 | 146.5 | -0.66 | 6.8e-01n | 148.3 | 151.4 | 3.07 | 1.6e-01 n | 146.0 | 141.4 | -4.60 | 5.2e-02 n | 4.5e-07 * |
| ***Lipoprotein Particles nmol/L*** |  |  |  |  |  |  |  |  |  |  |  |  |  |
| Total VLDL | 174.7 | 156.3 | -18.35 | 3.4e-05 * | 173.5 | 156.3 | -17.25 | 4.9e-03 n | 175.9 | 156.4 | -19.51 | 2.4e-03 n | 6.4e-01 n |
| Large VLDL | 26.8 | 21.61 | -5.23 | 4.8e-07 * | 26.04 | 20.21 | -5.83 | 2.4e-05 * | 27.68 | 23.09 | -4.58 | 3.2e-03 n | 2.6e-01 n |
| Medium VLDL | 71.8 | 62.79 | -9.04 | 1.4e-05 * | 71.30 | 61.58 | -9.72 | 6.7e-04 * | 72.39 | 64.06 | -8.33 | 6.1e-03 n | 5.3e-01 n |
| Small VLDL | 76.02 | 71.94 | -4.08 | 1.7e-02n | 76.17 | 74.47 | -1.70 | 4.8e-01 n | 75.85 | 69.26 | -6.60 | 6.1e-03 n | 1.7e-02 n |
| IDL | 173.9 | 162.9 | -11.01 | 2.4e-03n | 173.4 | 165.5 | -7.86 | 1.3e-01 n | 174.6 | 160.2 | -14.33 | 5.4e-03 n | 1.0e-01 n |
| Total LDL | 1,539 | 1,476 | -63.62 | 2.3e-02n | 1,542 | 1,527 | -15.59 | 6.9e-01 n | 1,537 | 1,422 | -114.43 | 4.1e-03 n | 6.4e-04 * |
| Large LDL | 794.9 | 796.0 | 1.10 | 9.5e-01n | 804.2 | 860.7 | 56.49 | 2.4e-02 n | 785.2 | 727.7 | -57.51 | 2.0e-02 n | 2.8e-08 * |
| Medium LDL | 233.6 | 217.3 | -16.31 | 1.6e-02n | 232.5 | 216.5 | -16.01 | 9.2e-02 n | 234.9 | 218.2 | -16.62 | 8.2e-02 n | 9.3e-01 n |
| Small LDL | 191.7 | 169.5 | -22.17 | 3.9e-03n | 190.0 | 162.2 | -27.85 | 8.2e-03 n | 193.4 | 177.3 | -16.17 | 1.5e-01 n | 1.5e-01 n |
| Very Small LDL | 319.2 | 292.9 | -26.23 | 2.8e-03n | 315.5 | 287.3 | -28.22 | 1.8e-02 n | 323.1 | 298.9 | -24.13 | 6.2e-02 n | 6.9e-01 n |
| Total HDL | 25.5 | 24.55 | -1.00 | 4.2e-03n | 25.46 | 25.01 | -0.46 | 3.5e-01 n | 25.62 | 24.06 | -1.57 | 1.7e-03 n | 3.3e-03 n |
| Large HDL | 7.07 | 7.02 | -0.05 | 7.2e-01n | 7.01 | 7.24 | 0.23 | 1.7e-01 n | 7.12 | 6.79 | -0.33 | 7.7e-02 n | 2.4e-06 * |
| Small HDL | 18.48 | 17.53 | -0.95 | 1.2e-04 * | 18.45 | 17.77 | -0.68 | 5.4e-02 n | 18.50 | 17.27 | -1.24 | 3.4e-04 n | 4.9e-02 n |
| LDL Peak Diameter Å | 221.2 | 222.6 | 1.32 | 1.0e-03n | 221.5 | 223.8 | 2.25 | 4.5e-05 * | 221.0 | 221.3 | 0.34 | 5.6e-01 n | 7.1e-07 * |
| Fasting Glucose mg/dL | 98.43 | 95.66 | -2.77 | 6.5e-06 * | 98.43 | 96.13 | -2.31 | 9.6e-03 n | 98.43 | 95.17 | -3.26 | 1.1e-04 n | 2.8e-01 n |
| Fasting Insulin µU/L | 15.33 | 12.44 | -2.88 | 9.1e-06 * | 14.94 | 11.85 | -3.09 | 7.6e-07 * | 15.74 | 13.07 | -2.66 | 2.2e-02 n | 6.9e-01 n |
| HOMA-IR | 3.78 | 2.97 | -0.80 | 1.7e-06 * | 3.69 | 2.86 | -0.83 | 7.9e-07 * | 3.88 | 3.10 | -0.78 | 8.6e-03 n | 8.7e-01 n |
| LC: low-carbohydrate diet; LF: low-fat diet. * Statistically significant at the Bonferroni-adjusted threshold of P<. 001 | | | | | | | | | | | | | |

**Table S5. Associations of change in BMI with changes in laboratory measurements**

**from baseline to 6 months adjusted for age, sex and diet group**

| ***Changes in:*** | **beta (CI)** | **P value** | **R2** |
| --- | --- | --- | --- |
| sLDLR ** | .817 (.645, .989) | 4.6e-19 * | 0.21 |
| ***Lipids and Apoproteins mg/dL*** |  |  |  |
| Cholesterol | 1.37 (.289, 2.44) | 1.3e-02 | 0.05 |
| Triglycerides | 5.91 (3.90, 7.92) | 1.4e-08 * | 0.12 |
| LDL-C | .178 (-.760, 1.12) | 7.1e-01 | 0.05 |
| HDL-C | .0062 (-.278, .291) | 9.7e-01 | 0.06 |
| Apo B | 1.25 (.624, 1.88) | 1.1e-04 | 0.05 |
| Apo A1 | .218 (-.527, .963) | 5.6e-01 | 0.07 |
| ***Lipoprotein Particles nmol/L*** |  |  |  |
| Total VLDL | 4.64 (2.29, 7.00) | 1.2e-04 * | 0.04 |
| Large VLDL | 1.44 (.905, 1.98) | 1.9e-07 * | 0.08 |
| Medium VLDL | 2.24 (1.14, 3.33) | 7.0e-05 * | 0.04 |
| Small VLDL | .966 (-.0551, 1.99) | 6.4e-02 | 0.02 |
| IDL | 3.27 (1.32, 5.22) | 1.1e-03 * | 0.03 |
| Total LDL | 28.3 (14.2, 42.5) | 9.5e-05 * | 0.06 |
| Large LDL | 8.05 (-1.99, 18.1) | 1.2e-01 | 0.08 |
| Medium LDL | 5.03 (1.32, 8.73) | 7.9e-03 | 0.03 |
| Small LDL | 5.48 (1.45, 9.52) | 7.9e-03 | 0.04 |
| Very Small LDL | 9.77 (4.79, 14.8) | 1.3e-04 * | 0.05 |
| Total HDL | .317 (.130, .503) | 9.1e-04 * | 0.04 |
| Large HDL | .0064 (-.0522, .0649) | 8.3e-01 | 0.05 |
| Small HDL | .310 (.173, .448) | 1.2e-05 * | 0.05 |
| LDL Peak Diameter Å | -.364 (-.549, -.180) | 1.2e-04 * | 0.12 |
| Fasting Glucose mg/dL | .865 (.435, 1.29) | 9.0e-05 * | 0.04 |
| Fasting Insulin µU/L | .910 (.376, 1.44) | 8.7e-04 * | 0.04 |
| HOMA-IR | .250 (.113, .387) | 3.8e-04 * | 0.04 |
| *** Statistically significant at the Bonferroni-adjusted threshold of P<.002  ** Measured as linearized Ct units as described in Methods | | | |

**Table S6. Associations of six-month change in sLDLR adjusted for age, sex and changes in BMI with changes in laboratory measurements for each diet**

|  | *Effect of changes in sLDLR among participants on a LC diet* | | | *Effect of changes in sLDLR among participants on a LF diet* | | | *P-value:*  *LF vs LC Diet* |
| --- | --- | --- | --- | --- | --- | --- | --- |
|  | beta (CI) | P value | R2 | beta (CI) | P value | R2 |  |
| Cholesterol | 1.45 (.636, 2.27) | 5.5e-04 | 0.06 | 1.73 (.977, 2.48) | 5.5e-04 * | 0.16 | 4.9e-02 |
| Triglycerides | 6.04 (4.62, 7.46) | 5.2e-15 * | 0.31 | 6.1 (4.89, 7.32) | 5.2e-15 * | 0.38 | 7.3e-01 n |
| LDL-C | .252 (-.504, 1.01) | 5.1e-01 | 0.04 | .266 (-.400, .931) | 5.1e-01 | 0.03 | 2.6e-01n |
| HDL-C | -.0064 (-.227, .214) | 9.5e-01 | 0.04 | .242 (.0357, .447) | 9.5e-01 | 0.06 | 1.9e-01 n |
| Apo B | 1.10 (.615, 1.59) | 1.3e-05 * | 0.08 | 1.06 (.633, 1.49) | 1.3e-05 * | 0.20 | 1.9e-03 |
| Apo A1 | .267 (-.311, .845) | 3.6e-01 | 0.05 | .705 (.171, 1.24) | 3.6e-01 | 0.10 | 7.3e-03 n |
| Total VLDL | 3.65 (1.65, 5.65) | 3.9e-04 * | 0.08 | 3.32 (1.83, 4.82) | 3.9e-04 * | 0.14 | 8.2e-01 n |
| Large VLDL | 1.17 (.796, 1.55) | 4.1e-09 * | 0.21 | 1.2 (.823, 1.58) | 4.1e-09 * | 0.21 | 8.8e-01 n |
| Medium VLDL | 2.04 (1.13, 2.95) | 1.4e-05 * | 0.11 | 1.68 (.975, 2.38) | 1.4e-05 * | 0.14 | 7.2e-01 n |
| Small VLDL | .435 (-.491, 1.36) | 3.6e-01 | 0.02 | .442 (-.183, 1.07) | 3.6e-01 | 0.05 | 4.3e-01 n |
| IDL-1 | 1.7 (.0397, 3.37) | 4.5e-02 | 0.02 | 1.37 (.0871, 2.66) | 4.5e-02 | 0.11 | 4.9e-01 n |
| Total LDL | 4.72 ( -7.34, 16.8) | 4.4e-01 | 0.01 | 4.31 ( -5.19, 13.8) | 4.4e-01 | 0.11 | 2.9e-01 n |
| Large LDL | -13.2 ( -21.9, -4.62) | 2.8e-03 | 0.06 | -13.1 ( -19.4, -6.85) | 2.8e-03 | 0.11 | 3.1e-01 n |
| Medium LDL | 3.03 (-.043, 6.11) | 5.3e-02 | 0.03 | .736 ( -1.85, 3.32) | 5.3e-02 | 0.06 | 6.7e-01 n |
| Small LDL | 7.8 (4.48, 11.1) | 6.0e-06 * | 0.11 | 6.69 (4.11, 9.28) | 6.0e-06 * | 0.14 | 9.0e-01 n |
| Very Small LDL | 7.13 (2.91, 11.4) | 1.0e-03 | 0.10 | 10 (7.00, 13.1) | 1.0e-03 | 0.22 | 1.3e-01 n |
| LDL Peak Diameter | -.342 (-.484, -.199) | 4.2e-06 * | 0.17 | -.438 (-.559, -.317) | 4.2e-06 * | 0.23 | 4.9e-01 n |
| Total HDL | .0223 (-.137, .181) | 7.8e-01 | 0.01 | .0646 (-.0622, .191) | 7.8e-01 | 0.07 | 2.4e-01 n |
| Large HDL | -.0061 (-.0568, .0445) | 8.1e-01 | 0.02 | -.0207 (-.0595, .0181) | 8.1e-01 | 0.03 | 7.2e-01 n |
| Small HDL | .0284 (-.0876, .144) | 6.3e-01 | 0.02 | .0853 (-.0091, .180) | 6.3e-01 | 0.10 | 1.5e-01 n |
| Fasting Glucose | -.165 (-.505, .175) | 3.4e-01 | 0.05 | -.066 (-.384, .252) | 3.4e-01 | 0.04 | 7.4e-01 n |
| Fasting Insulin | -.0602 (-.281, .161) | 5.9e-01 | 0.19 | .151 (-.359, .661) | 5.9e-01 | 0.01 | 8.8e-01 n |
| HOMA_IR | -.0214 (-.0798, .0369) | 4.7e-01 | 0.20 | .0358 (-.0948, .166) | 4.7e-01 | 0.02 | 8.2e-01 n |
| * Significant at the Bonferroni-adjusted threshold of P = .001 | | | | | | | |

**Table S7. Correlations between lipoprotein levels at baseline (upper panel) and their changes between baseline and 6 months (lower panel)**

| ***Baseline levels:*** | **Large VLDL** | **Medium VLDL** | **Small VLDL** | **IDL** | **Large LDL** | **Medium LDL** | **Small LDL** | **Very Small LDL** | **Large HDL** | **Small HDL** |
| --- | --- | --- | --- | --- | --- | --- | --- | --- | --- | --- |
| Large VLDL | 1.00 |  |  |  |  |  |  |  |  |  |
| Medium VLDL | 0.93 | 1.00 |  |  |  |  |  |  |  |  |
| Small VLDL | 0.54 | 0.75 | 1.00 |  |  |  |  |  |  |  |
| IDL | 0.57 | 0.75 | 0.92 | 1.00 |  |  |  |  |  |  |
| Large LDL | 0.05 | 0.21 | 0.60 | 0.56 | 1.00 |  |  |  |  |  |
| Medium LDL | 0.54 | 0.59 | 0.47 | 0.65 | 0.32 | 1.00 |  |  |  |  |
| Small LDL | 0.66 | 0.65 | 0.38 | 0.56 | -0.11 | 0.76 | 1.00 |  |  |  |
| Very Small LDL | 0.69 | 0.67 | 0.42 | 0.51 | -0.12 | 0.39 | 0.77 | 1.00 |  |  |
| Large HDL | 0.03 | 0.12 | 0.41 | 0.36 | 0.49 | 0.05 | -0.07 | 0.06 | 1.00 |  |
| Small HDL | 0.41 | 0.48 | 0.59 | 0.61 | 0.54 | 0.41 | 0.28 | 0.31 | 0.73 | 1.00 |
|  | | | | | | | | | | |
| ***Baseline to 6-month changes:*** | **Large VLDL** | **Medium VLDL** | **Small VLDL** | **IDL** | **Large LDL** | **Medium LDL** | **Small LDL** | **Very Small LDL** | **Large HDL** | **Small HDL** |
| Large VLDL | 1.00 |  |  |  |  |  |  |  |  |  |
| Medium VLDL | 0.85 | 1.00 |  |  |  |  |  |  |  |  |
| Small VLDL | 0.37 | 0.71 | 1.00 |  |  |  |  |  |  |  |
| IDL-1 | 0.35 | 0.62 | 0.85 | 1.00 |  |  |  |  |  |  |
| Large LDL | 0.01 | 0.19 | 0.52 | 0.54 | 1.00 |  |  |  |  |  |
| Medium LDL | 0.19 | 0.27 | 0.24 | 0.51 | 0.28 | 1.00 |  |  |  |  |
| Small LDL | 0.31 | 0.31 | 0.17 | 0.42 | -0.18 | 0.59 | 1.00 |  |  |  |
| Very Small LDL | 0.42 | 0.41 | 0.28 | 0.39 | -0.08 | 0.10 | 0.63 | 1.00 |  |  |
| Large HDL | 0.19 | 0.32 | 0.52 | 0.53 | 0.60 | 0.26 | 0.15 | 0.29 | 1.00 |  |
| Small HDL | 0.42 | 0.48 | 0.52 | 0.64 | 0.53 | 0.47 | 0.38 | 0.39 | 0.75 | 1.00 |

**Table S8. Associations of changes in the top 3 principal components (PCs) with changes in sLDLR, BMI, and diet assignment from baseline to 6 months**

**Table S8A. Relationships of changes in sLDLR to predicted changes in levels of the top 3 PCs:**

| Change in sLDLR | beta | CI-low | CI-high | p-value | R2 |
| --- | --- | --- | --- | --- | --- |
| PC1 | 0.109 | 0.068 | 0.150 | 3.0e-07 * | .056 |

| Change in sLDLR | beta | CI-low | CI-high | p-value | R2 |
| --- | --- | --- | --- | --- | --- |
| PC2 | 0.344 | 0.284 | 0.404 | 5.8e-26 * | .215 |

| Change in sLDLR | beta | CI-low | CI-high | p-value | R2 |
| --- | --- | --- | --- | --- | --- |
| PC3 | -0.091 | -0.172 | -0.009 | 2.9e-02 * | .010 |

Tested jointly, the 3 PCs explain 28% of the variance in mean change in sLDLR.

**Table S8B. Relationships of changes in BMI to predicted changes in levels of the top 3 PCs, adjusted for diet:**

| **Change in BMI** | **beta** | **CI-low** | **CI-high** | **p-value** | **R2** |
| --- | --- | --- | --- | --- | --- |
| PC1 | 0.092 | 0.050 | 0.133 | 1.6e-05 * | .054 |

| Change in BMI | 0 | CI-low | CI-high | p-value | R2 |
| --- | --- | --- | --- | --- | --- |
| PC2 | 0.100 | 0.031 | 0.168 | 4.7e-03 * | .032 |

| Change in BMI | beta | CI-low | CI-high | p-value | R2 |
| --- | --- | --- | --- | --- | --- |
| PC3 | -0.022 | -0.103 | 0.060 | 6.0e-01 | .015 |

Tested jointly, the 3 PCs, adjusted for diet, explain 7.0% of the variance in mean change in BMI.

**Table S8C. Odds of association of the predicted levels of the top 3 PCs with the low-carbohydrate diet vs odds of their association with the low-fat diet:**

| LC Diet | Odds Ratio | CI-low | CI-high | p-value |
| --- | --- | --- | --- | --- |
| PC1 | 1.09 | 0.997 | 1.18 | 5.8e-02 * |

| LC Diet | Odds Ratio | CI-low | CI-high | p-value |
| --- | --- | --- | --- | --- |
| PC2 | 0.682 | 0.582 | 0.799 | 2.1e-06 * |

| LC Diet | Odds Ratio | CI-low | CI-high | p-value |
| --- | --- | --- | --- | --- |
| PC3 | 1.04 | 0.887 | 1.23 | 6.0e-01 * |

PCs were calculated using lipoprotein fractions only. All values are based on z-score standardized differences between baseline and 6 months, Each analysis is a univariate test against each PC individually.

*Statistical significance based on a Bonferroni-adjusted threshold of p = .006.

LC: low-carbohydrate diet; LF: low-fat diet
